# A Comprehensive Review of Kimura Disease

**DOI:** 10.1007/s12105-025-01812-z

**Published:** 2025-06-23

**Authors:** Ian T. Lagerstrom, David T. Danielson, Jeannie M. Muir, Robert D. Foss, Aaron Auerbach, Nadine S. Aguilera

**Affiliations:** 1 Department of Pathology, Walter Reed National Military Medical Center, Bethesda, MD USA; 2 Department of Pathology, Uniformed Services University of the Health Sciences, Bethesda, MD USA; 3 Joint Pathology Center, Silver Spring, MD USA; 4 University of Virginia Health System, Charlottesville, VA USA

**Keywords:** Eosinophilia, Kimura disease, Hematopathology, Head and neck pathology

## Abstract

**Purpose:**

Kimura disease (KD) is a rare, chronic inflammatory disorder that primarily affects the head and neck regions, often mimicking neoplastic conditions. This study aims to provide a comprehensive review of KD, focusing on its clinical presentation, diagnostic challenges, optimal management strategies, and primary histopathologic differential diagnosis.

**Methods:**

A systematic review of literature was conducted using PubMed, Scopus, and Google Scholar databases. We analyzed case reports, retrospective studies, and clinical trials published in English. We extracted data on epidemiology, clinical presentation, laboratory findings, histologic features, current understanding of the pathogenesis, treatment, and prognosis.

**Results:**

KD predominantly affects young Asian males, presenting with painless subcutaneous masses, peripheral eosinophilia, and elevated serum IgE levels. Histopathology reveals lymphoid follicular hyperplasia with eosinophilic infiltration. Biopsy is required for diagnosis. The pathogenesis of KD is poorly understood, but recent studies have elucidated some potentially important mechanisms of the disease. Treatment options include systemic corticosteroids, surgical excision, radiotherapy, and cytotoxic therapies, with recurrence rates varying among modalities.

**Conclusion:**

KD remains a diagnostic challenge due to its overlapping features with a variety of neoplastic and non-neoplastic conditions. While corticosteroids offer temporary relief and can be useful in cases with renal involvement, surgical excision remains the most definitive treatment. Future research should focus on targeted therapies to improve long-term disease control and reduce recurrence.

## Introduction

Kimura disease (KD) is a rare, benign, chronic inflammatory condition of uncertain etiology, predominantly affecting subcutaneous tissues, salivary glands, and lymph nodes in the head and neck region [1]. First described in 1937 by Kimm and Szeto [2] and later named by Kimura et al. in 1948 [3], this disease is characterized by painless subcutaneous nodules, peripheral blood eosinophilia, and elevated serum IgE levels [4]. It predominantly affects young to middle-aged men, and is endemic to regions such as Japan, China, and Southeast Asia. It is occasionally reported in other populations [1, 3, 4]. KD has clinical and histomorphologic overlap with both benign and malignant lesions that are commonly localized to the head and neck, making familiarization with the disease process and accurate diagnosis important.

The first account of KD was in 1937 when Kimm and Szeto described a unique inflammatory lesion in the head and neck [2]. In 1948, Kimura et al. expanded on this description in their paper titled “On the Unusual Granulation Combined with Hyperplastic Changes of Lymphatic Tissue,” coining the term eosinophilic hyperplastic granuloma [3]. In 1969, Wells and Whimster described an ill-defined subcutaneous inflammatory and vascularized process associated with peripheral and tissue eosinophilia, introducing the name subcutaneous angiolymphoid hyperplasia with eosinophilia (ALHE) [5].

Wells and Whimster believed that KD and ALHE represented the same disease process with the early stages of the disease presenting as epithelioid vascular change with a lack of lymphoid aggregates that progressed to mature vessels and lymphoid hyperplasia. By the early 1980s, it became increasingly clear that KD and ALHE represented different disease processes due to their distinct clinical and histomorphological features [6–8]. From this point onwards, KD was recognized as a distinct entity, separating it from other vascular proliferations and inflammatory processes. The term ALHE persisted in the literature and in usage by pathologists to denote epithelioid hemangioma (EH).

Despite being recognized as a unique entity, KD has undergone little diagnostic evolution since its inception, and the pathogenesis of the disease remains unclear. This review provides a detailed discussion of the clinical presentation, laboratory findings, histologic features, current understanding of the pathogenesis, treatment, prognosis, and differential diagnosis of KD.

## Clinical Presentation and Laboratory Findings

**Fig. 1:**
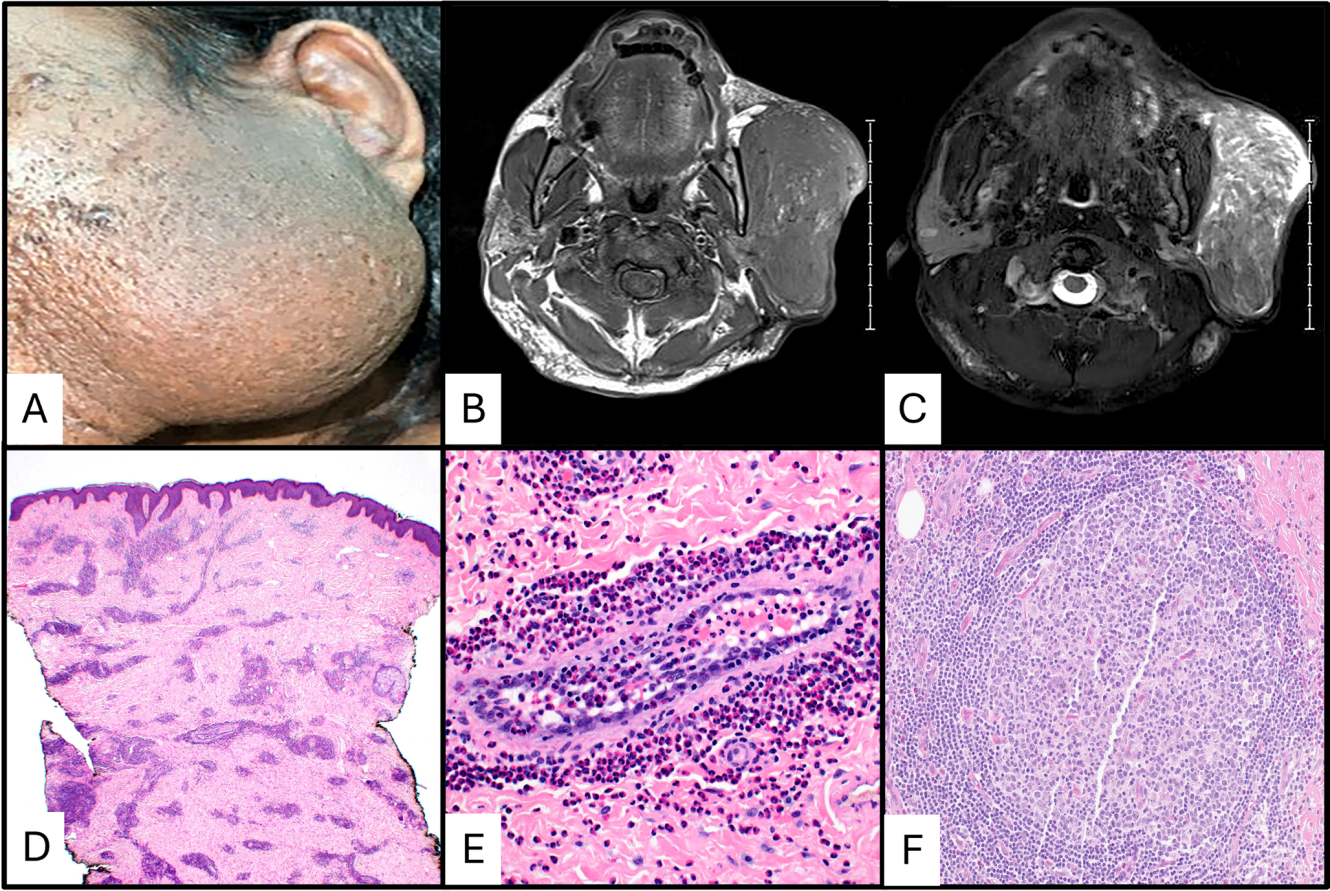
41-year-old male (Marshal Islander/Asian/Pacific Islander) presented with a large, recurrent parotid mass eight years after initial surgical excision (**a**). The patient’s serum IgE level was 5783 IU/ml (normal range 6-495 IU). Magnetic resonance imaging revealed a large T1 isointense mass in the left parotid region (**b**). The mass is hyperintense on the T2-weighted image (**c**). An additional lesional focus is present in the postauricular region. The mass on punch biopsy (**d**) showed a deeply seated lesion composed of a mixed inflammatory infiltrate with prominent eosinophilia and indistinct margins. Eosinophilic vasculitis (**e**) and subcutaneous secondary follicles with surrounding eosinophils (**f**) were present

KD presents as painless, ill-defined subcutaneous nodules or mass-like lesions (Fig. 1a) that occur in the head and neck region (greater than 75% of cases) or occasionally localized to the extremities [4, 9–12]. Lymphadenopathy is present in most cases, and salivary gland involvement is seen in a smaller subset of cases [1, 4, 10, 11]. Systemic symptoms are uncommon, but pruritus, eczematous rashes, and other skin changes occasionally occur [4, 13]. Other systemic symptoms, such as fever, weight loss, and night sweats, are uncommon but have been reported [13–15]. When these uncommon systemic symptoms are present, neoplastic and infectious etiologies are important clinical considerations.

Magnetic resonance imaging (MRI) can be a useful adjunct in the evaluation of KD lesions. They are mostly circumscribed, are characteristically isointense on T1-weighted images (Fig. 1b), and are T2 hyperintense (Fig. 1c). Post contrast enhancement most frequently shows a heterogeneous pattern [16, 17]. Laboratory findings can aid in making the diagnosis of KD with serum IgE levels and peripheral eosinophilia being the most useful. Serum IgE levels are elevated in greater than 90% of cases, commonly many times above the upper limit of normal, and there is frequently moderate to marked peripheral eosinophilia [4, 13]. Markers of inflammation may also be elevated [18]. Interestingly, a small portion of cases are associated with proteinuria and nephrotic syndrome with a wide range of pathologies on kidney biopsies reported to include minimal change disease, focal segmental glomerulosclerosis, IgA nephropathy, membranous nephropathy, and membranoproliferative glomerulonephritis [9, 19–22]. Although laboratory findings can help in the diagnosis, a biopsy of the masses associated with KD remains the cornerstone for diagnosis, as it must be differentiated from both lymphomas and other inflammatory and reactive etiologies.

### **Histologic Features and Immunohistochemistry**

**Fig. 2:**
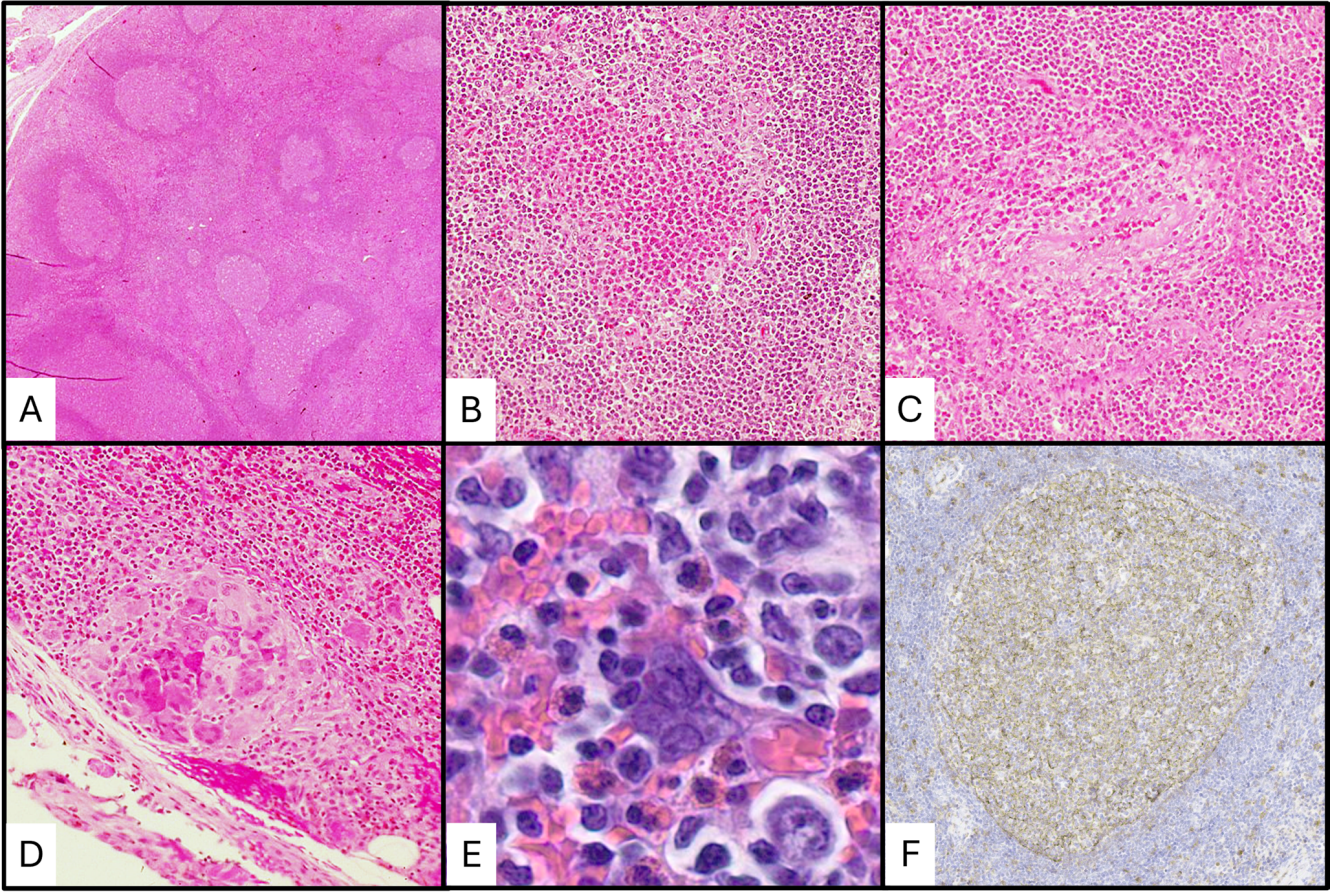
Histopathologic features of Kimura disease in a lymph node. (**a**) Low power showing follicle lysis. (**b**) High power showing an eosinophilic abscess. (**c**) High power showing eosinophilic vasculitis. (**d**) High power showing an eosinophilic granuloma. (**e**) High power showing a Warthin-Finkeldy polykaryocyte. (**f**) IgE reticular staining in a germinal center

Skin manifestations and lymph node involvement by KD show consistent features (Figs. 1d and f and 2). Florid follicular hyperplasia with preserved nodal architecture, diffuse interfollicular eosinophilic infiltrates, eosinophilic microabscesses, and prominent postcapillary venule proliferation with perivenular sclerosis are the most consistent features [4]. Other frequent findings include stromal sclerosis, vascularization of germinal centers, eosinophilic folliculitis, germinal center proteinaceous deposits, and polykaryocytes within germinal centers and/or interfollicular areas [4, 23, 24]. Germinal center necrosis is uncommon.

Immunohistochemistry (IHC) demonstrates germinal centers with typical IHC profiles seen in reactive follicular hyperplasia including immunoreactivity for CD10, CD20, CD79a, BCL-6, and PAX5 and absent immunoreactivity for BCL2 [4, 25]. The mantle zones are well formed in KD and demonstrate a mature B-cell immunoprofile [18]. Kappa and lambda light chain staining will show a polytypic pattern. IgE reticular staining within the germinal centers is a classic IHC feature in nearly all cases (Fig. 2f); although, this finding is not specific and is present in other reactive lymphadenopathies [4, 25, 26].

## Pathogenesis

The exact etiology of KD is not known. However, it is likely that T helper (Th) 2 cells and Th2 associated cytokines play an important role in the pathogenesis of KD. Th2 cells release interleukin (IL)-4, IL-5, and IL-13 [27]. IL-4 drives the immunoglobulin (Ig) class switching to IgG1 and IgE [27]. Increased levels of IL-5 stimulate eosinophil development in bone marrow and survival in the blood [28]. IL-4 and IL-13 also act to increase the production of C-C Motif Chemokine Ligand (CCL) 5, CCL11, CCL24, and CCL26, which enhance chemotaxis of eosinophils to tissues [29–32].

The percentage of Th2 cells and the Th2/Th1 ratio in the peripheral blood is significantly increased compared to healthy controls, and the number for Th2 cells in KD correlates directly with the serum IgE titer [33]. Similarly, stimulated peripheral blood T-cells from individuals with KD show a higher expression of Th2 cytokines, including IL-3, IL-4, IL-5, IL-13, and granulocyte-macrophage colony stimulating factor, compared to healthy controls [34]. Moreover, multicolor immunofluorescence staining of formalin-fixed, paraffin-embedded sections has shown that KD lesions are rich in Th2 cells as well as IgE and IL-4 positive mast cells [35].

An increase in Th2 cells and related cytokines may explain the tissue and peripheral eosinophilia and increased serum IgE that is seen in KD. IgE mediated mast cell recruitment, activation, and degradation may explain common symptoms seen in KD, including skin rashes and pruritus. The exact mechanism that leads to an increase in the Th2-mediated immune response in KD is not known. It is possibly the result of injury or inflammation that leads to exposure of self-antigens. Dysregulation of the Th2-mediated immune response plays a role in various autoimmune conditions [27, 36–40].

## **Prognosis and Treatment**

KD is considered a benign condition with no risk of metastasis [41]. The lesions of KD can be locally aggressive and may have local recurrence. Surgery is the primary treatment method, but radiation, systemic immunosuppression, and chemotoxic therapies have been employed with variable success [11, 41, 42]. In a meta-analysis by Lee et al., the recurrence rate for surgical excision was 30.5%, for systemic immunosuppression was 45%, and for radiotherapy was 60% [11]. Combinations of these treatment modalities have been employed to attempt to reduce recurrence and limit the need for prolonged systemic immunosuppression.

Adjuvant radiotherapy following surgery may reduce the risk of local recurrence compared to surgery alone. Surgical excision and adjuvant radiotherapy has been shown to reduce recurrence compared to surgery alone in two studies by Ye et al. [42, 43]. In a different study, no significant difference was found between the recurrence rates of radiotherapy and non-radiotherapy groups (*p* = 0.23) [44]. Further studies are required to determine the efficacy of radiotherapy in reducing the rates of local recurrence.

Proteinuria has been reported in 12–16% of patients with KD, with 60–78% of these patients developing nephrotic syndrome [45]. Steroids have treated the renal disease associated with KD with success in multiple reported cases [19–21, 46, 47]. Although recurrence risk may be higher with the use of steroids alone for treatment of KD, there may be a benefit for the use of steroids in cases with associated renal disease.

Targeted therapies have the potential to be more effective than traditional therapies with fewer systemic and off-target effects. Multiple case reports of successful treatment of Kimura disease with dupilumab have been published [48–52]. Dupilumab is a monoclonal antibody that inhibits the signaling of IL-4 and IL-13, two key cytokines involved in the Th2 immune response. It works by binding to the shared alpha subunit (IL-4Rα) of the IL-4 and IL-13 receptor complexes, preventing the release of proinflammatory cytokines, chemokines, nitric oxide, and IgE [53].

Dupilumab may be a good treatment option for KD because the disease is characterized by a Th2-dominated immune response with elevated levels of IL-4, IL-5, IL-10, and IL-13, which play key roles in its pathogenesis [33–35]. By blocking the signaling of IL-4 and IL-13 through the IL-4Rα receptor, dupilumab can directly inhibit Th2-mediated inflammation, reducing eosinophil recruitment and tissue damage. This targeted mechanism addresses the underlying immune dysregulation, offering a rational and potentially effective treatment approach.

Bulk-RNA sequencing has demonstrated that the extracellular-regulated kinase/mitogen-activated protein kinase (Erk/MAPK) signaling pathway is over-activated in the eosinophils of patients with KD compared to healthy controls [54]. The Erk/MAPK signaling pathway is essential for eosinophil activation, degranulation, and migration, supporting that overactivation of the pathway may play an integral role in the pathogenesis of Kimura disease [55, 56]. The exact mechanism leading to Erk/MAPK signaling pathway over-activation in KD is unknown, but this provides an option for targeted therapy, such as MAPK inhibitors. Further understanding of the pathogenesis of KD may provide more options for targeted therapy in the future.

## Differential Diagnosis

The differential diagnosis of KD encompasses a variety of conditions that share clinical and histopathologic features with KD. These can be broadly categorized into non-neoplastic and neoplastic entities. Accurate differentiation is crucial for appropriate management and prognosis. To facilitate comparison and highlight key elements, Table 1 provides an overview of KD and its main differential diagnoses.

**Table 1:** Key clinical and histopathologic elements of Kimura disease and its differential diagnoses

Entity	Clinical Presentation	Histopathologic Features	Immunohistochemistry
Kimura Disease	Subcutaneous masses in head/neck, lymphadenopathy, pruritus, peripheral eosinophilia, elevated serum IgE	Follicular hyperplasia, eosinophils, eosinophilic folliculolysis and microabscesses, postcapillary venule proliferation, proteinaceous deposits, Warthin-Finkeldy polykaryocytes	IgE reticular staining within germinal centers
Epithelioid Hemangioma	Subcutaneous masses or papules in head/neck, pruritus, rare peripheral eosinophilia	Vascular proliferation with hobnailed endothelial cells, eosinophils	FOSB nuclear expression in endothelial cells
Nodal T Follicular Helper Cell Lymphoma, Follicular Type	Generalized lymphadenopathy, advanced-stage disease	Effacement of lymph node architecture, follicular pattern, intermediate-sized lymphocytes, eosinophils	CD3+, CD4+, strong PD1+, and one other T follicular helper cell marker
Classic Hodgkin Lymphoma	Nodal, supradiaphragmatic masses, B symptoms	Effacement of lymph node architecture, Hodgkin-Reed-Sternberg (HRS) cells, eosinophils	CD15+, CD30+, weak PAX5 + in HRS cells
Langerhans Cell Histiocytosis	Unifocal or multifocal, cutaneous lesions and lymphadenopathy in head/neck, often in children	Effacement of lymph node architecture, histiocytes with grooved nuclei, eosinophils	CD1a+, S100+, and CD207 + in histocytes
Reactive Lymphadenopathy	Lymphadenopathy, variable systemic symptoms	Variable histology (follicular hyperplasia, paracortical/interfollicular hyperplasia, sinus histiocytosis), variable eosinophils	Polyclonal B- and T-cells, no specific markers
IgG4-Related Disease	Multisystem involvement, lymphadenopathy, elevated serum IgG4	Fibrosis, plasma cell infiltrates, follicular hyperplasia	Elevated IgG4 + plasma cells

Among the differential diagnoses, EH is the closest mimic of KD. Clinically, both conditions can manifest as subcutaneous masses in the head and neck region with associated pruritus [57, 58]. Peripheral blood eosinophilia may be present, but elevation of serum IgE is uncommon [59]. While KD tends to present at a younger age compared to EH, the overall clinical presentation can be quite similar, especially for the subcutaneous presentation of EH, making biopsy and histologic evaluation critical [60].

**Fig. 3:**
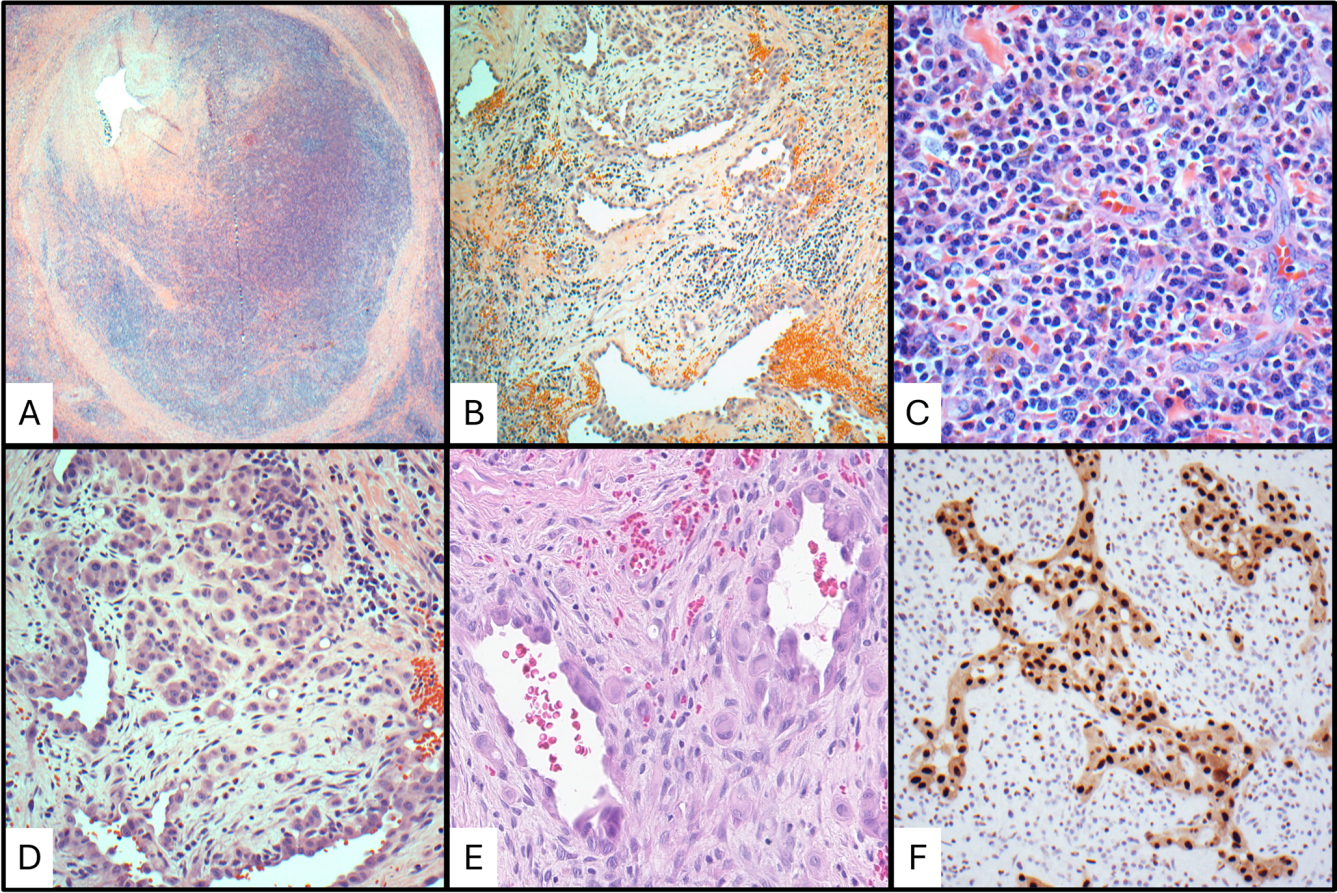
Differential diagnosis of epithelioid hemangioma. (**a**) Low power showing a subcutaneous nodule. (**b**) Low power of vessels showing plump endothelial cells (epithelioid change). (**c**) High power of mixed inflammation with eosinophils and vascular hyperplasia. (**d**) High power of prominent plump endothelial cells. (**e**) ERG expression in the endothelial cells

Histologically, EH shows a proliferation of variably sized vessels lined by cuboidal endothelial cells described as “hobnailed” and epithelioid with abundant clear to eosinophilic and vacuolated cytoplasm (Fig. 3) [57]. FOSB nuclear positivity in the endothelial cells of EH has been reported [61], likely due to oncogenic *FOS* rearrangements [62, 63]. Mitotic activity and atypia are not usually present. The proliferation of vessels is accompanied by a dense inflammatory infiltrate composed of lymphocytes and eosinophils. The lymphocytes are mostly T-cells, but lymphoid follicles composed of B-cells can be seen. In early lesions, the vascular component with epithelioid endothelial cells is predominant, and the inflammatory infiltrate increases as the disease progresses [57, 64].

Cases of EH with prominent eosinophilic infiltrate and lymphoid follicles can have significant histologic overlap with KD. While KD can have numerous post capillary venules, the endothelial cells do not appear hobnailed or epithelioid, like in EH [4]. Other frequently seen histologic features of KD, including eosinophilic folliculolysis, germinal center vascularization, germinal center proteinaceous deposits, and polykaryocytes, are absent [4, 7].

Nodal T follicular helper (TFH) cell lymphomas (nTFHL) are another important consideration with a lesser degree of clinical and histological overlap compared to EH. These are mature T-cell neoplasms with morphologic, immunophenotypic, and molecular features of TFH cells. TFH cells are a type of CD4 + T-cell that play a role in memory B-cell development and primarily reside in secondary follicles of normal lymph nodes. The clinical presentation does not mimic KD as closely as EH. Patients commonly have serum markers of inflammation, such as an increased erythrocyte sedimentation rate, and may show non-clonal hypergammaglobulinemia. IgE elevations are not seen, but peripheral eosinophilia and skin rashes can occur [65].

**Fig. 4:**
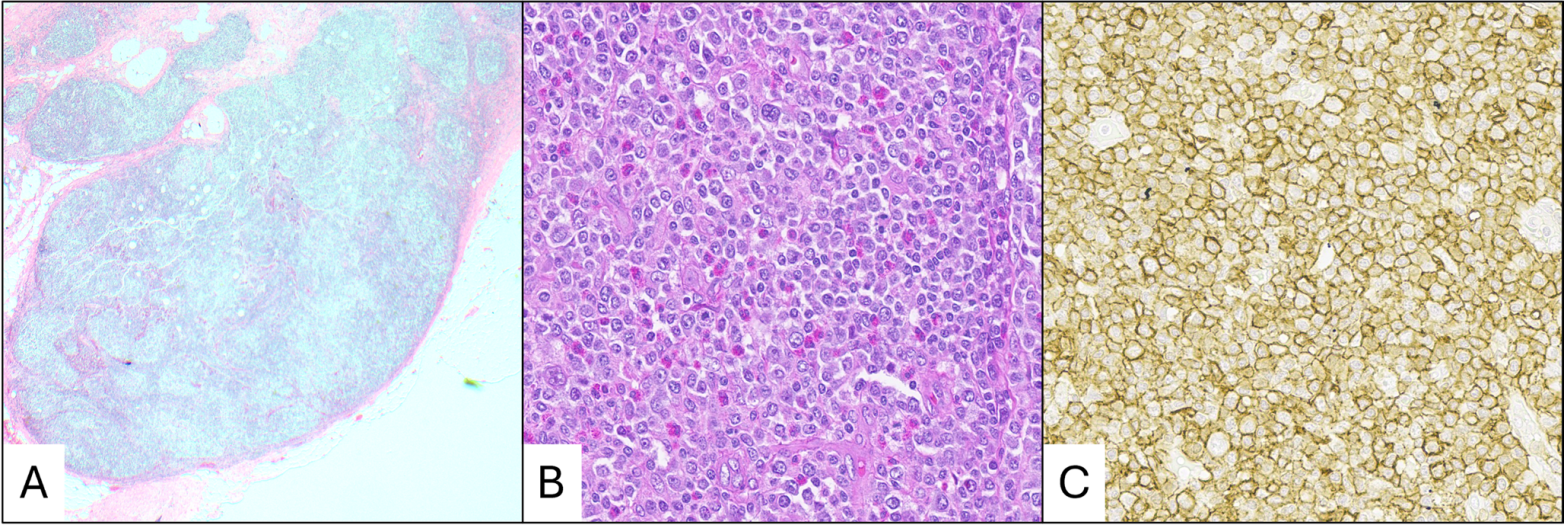
Differential diagnosis of nodal T follicular helper cell lymphoma. (**a**) Low power image of nodal T follicular helper cell lymphoma, NOS. (**b**) High power showing characteristic features of neoplastic T-cells with background eosinophils. (**c**) PD1 expression in the neoplastic T-cells

nTFHL, follicular type (nTFHL-F) demonstrates a follicular lymphoma-like pattern and has the most histopathologic overlap with KD. In nTFHL-F, the lymph node architecture is effaced by a nodular proliferation of intermediate-sized lymphocytes with moderate to abundant clear to eosinophilic cytoplasm with nuclei that are round to mildly irregular (Fig. 4) [65]. Eosinophils are often present. Assessment of a wide range of immunophenotypic markers for T-cell origin (CD2, CD3, CD4, CD5, CD7, and CD8) and TFH cell origin (CD10, ICOS, PD1, CXCL13) is useful. Expression of at least two markers of TFH origin, including strong PD1, is required for a diagnosis [65].

Compared to the expanded follicles in nTFHL-F, the follicles in KD are benign reactive follicles composed primarily of B-cells and have the typical expression of BCL-6 and CD10. Expression of BCL-6 and CD10 by neoplastic T-cells in nTFHL-F can occur, but should not be confused with normal follicular B-cells [65]. Additionally, a t(5, 9)(q33; q22), resulting in a *ITK-SYK* fusion transcript, has been identified in cases of nTFHL-F [66], as well as mutations in genes such as TET2, DNMT3A, IDH2, and RHOA [67, 68]. In cases of KD and nTFHL-F with a high degree of histopathologic overlap, the use of IHC and molecular studies can help resolve the differential.

Other neoplastic entities characterized by an eosinophilic infiltrate on histology can occur in the head and neck, to include classic Hodgkin lymphoma (CHL) and Langerhans cell histiocytosis (LCH). While these entities do not pose a diagnostic challenge, we will discuss them briefly due to some overlapping morphologic features. CHL typically presents as a supradiaphragmatic nodal based disease, frequently involving the cervical lymph nodes, although primary extranodal disease can rarely occur [69, 70]. Peripheral blood eosinophilia is uncommon in CHL (15% of cases) and an elevated IgE is rarely seen [71, 72].

**Fig. 5:**
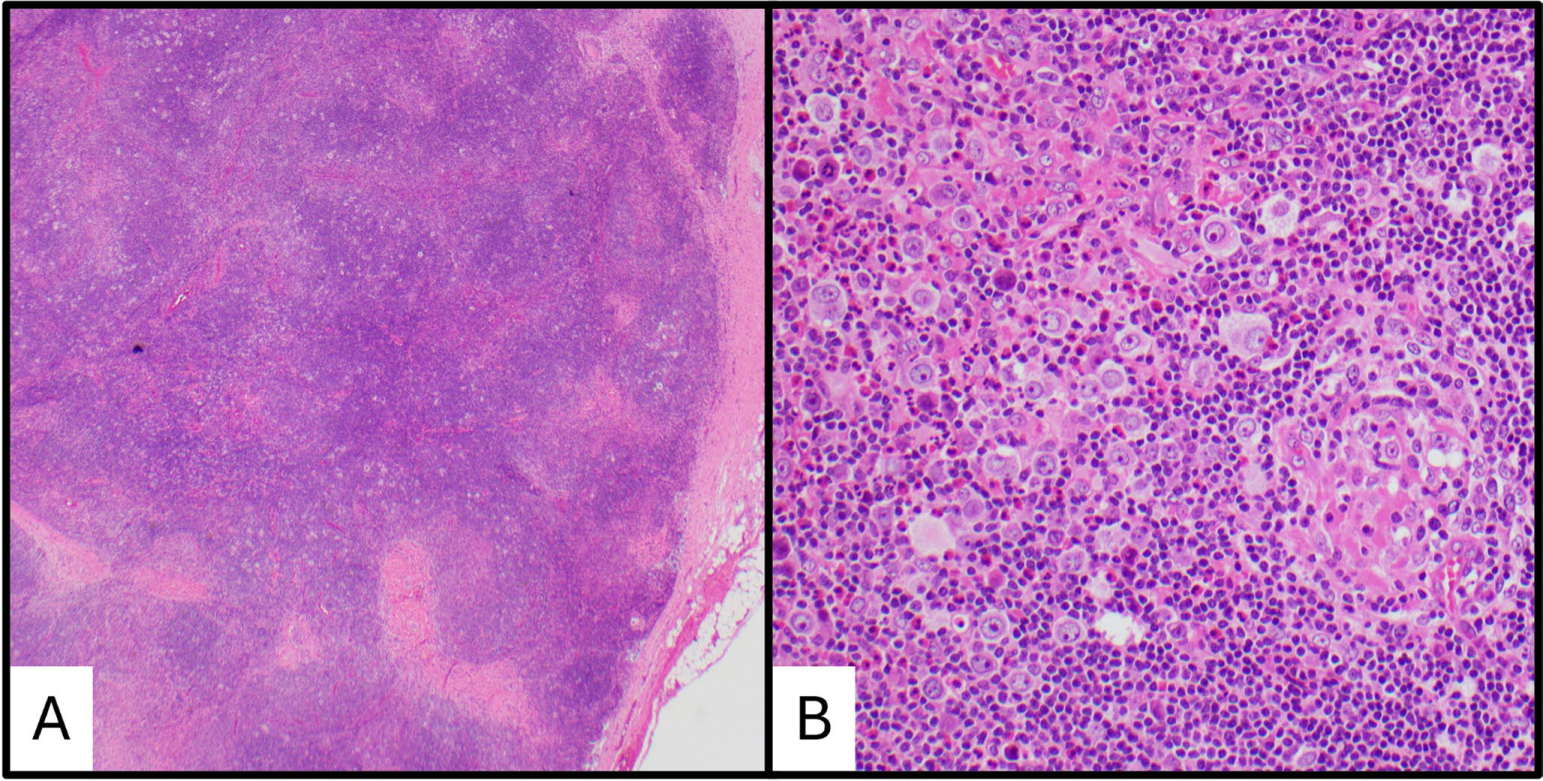
Differential diagnosis of classic Hodgkin lymphoma. (**a**) Low power showing effacement of lymph node architecture. (**b**) Intermediate power showing Hodgkin-Reed-Sternberg cells and numerous eosinophils

CHL is characterized by the presence of Hodgkin-Reed-Sternberg (HRS) cells (multinucleate) and Hodgkin cells (mononuclear) with a background inflammatory infiltrate of histiocytes, plasma cells, and eosinophils (Fig. 5). Immunoblasts in KD can mimic Hodgkin cells, but KD immunoblasts are typically smaller and do not have the prominent red nucleolus seen in Hodgkin cells. Further, immunoblasts will not be multinucleated, helping to differentiate from HRS cells. While sclerosis is often present in KD, sclerotic bands like those seen in nodular sclerosis CHL should not be [4].

LCH is a monoclonal neoplasm of myeloid dendritic cells that primarily occurs in children, but can occur at any age [69, 73]. LCH can be unifocal or multifocal and can involve bone, skin, lymph nodes, and multiple organs. In the head and neck, LCH commonly presents as cutaneous lesions or as cervical lymphadenopathy [69]. Cutaneous lesions can present as seborrheic dermatitis or eczematous eruptions, most commonly on the scalp and trunk [73].

**Fig. 6:**
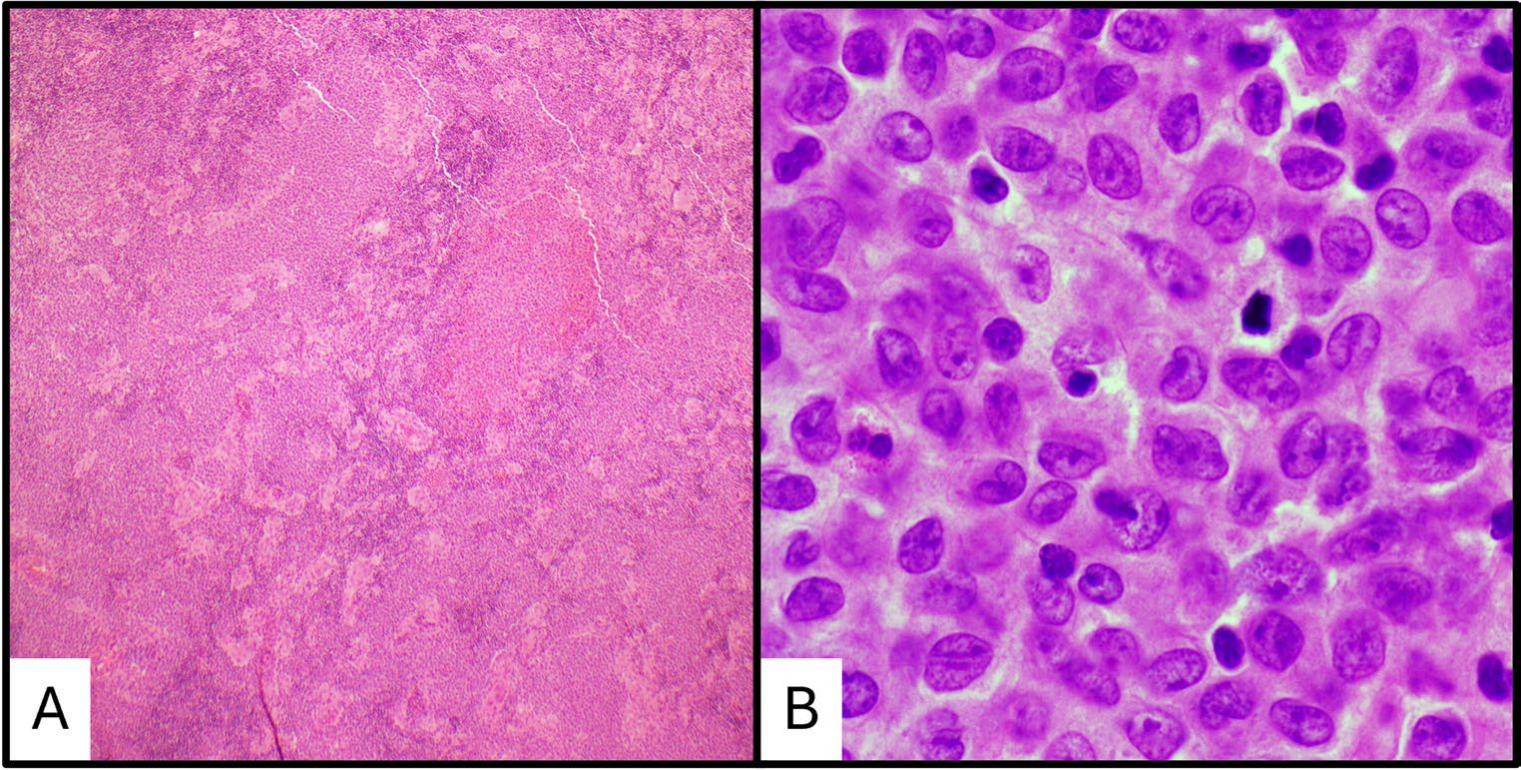
Differential diagnosis of Langerhans histiocytosis. (**a**) Low power showing effacement of lymph node architecture. (**b**) High power showing Langerhans cells with characteristic nuclear grooves and eosinophils

Lymph nodes involved by LCH show effacement of normal lymph node architecture with increased eosinophils (Fig. 6a). Follicular hyperplasia is not a common feature. The histiocytes in LCH have moderate to abundant pale pink cytoplasm and round to oval nuclei with complex contours and longitudinal grooves (Fig. 6b) and are immunoreactive for CD1a, S100, CD207 (langerin) and CD68 [73]. The Warthin-Finkeldy polykaryocytes seen in KD have morphologic similarities with the histiocytes in LCH, but these polykaryocytes demonstrate a T-cell phenotype, expressing CD43 and CD3. A BRAF V600E mutation is seen in approximately 36–70% of LCH cases [74], which is not seen in KD.

Among non-neoplastic conditions, reactive lymphadenopathies can present with follicular hyperplasia and varying degrees of eosinophilic infiltration. However, these are generally associated with identifiable etiologies such as infections, autoimmune disorders, or allergies [75], and lack the specific histopathologic hallmarks of KD, such as eosinophilic folliculolysis and Warthin-Finkeldy polykaryocytes.

Allergies can lead to lymphadenopathy and peripheral eosinophilia due to a Th2-mediated immune response [76]. This specific etiology of reactive lymphadenopathy can have significant clinicopathologic overlap with KD. Clinical history and allergy testing may be prudent in such cases before initiating treatment. Other specific etiologies of follicular hyperplasia, such as Castleman disease, toxoplasmosis, syphilis, and HIV-associated lymphadenopathy, have well-characterized features [77]. These diseases are not likely to be in the differential diagnosis of KD as they lack the other classic features of KD.

Rare cases of KD have been reported with IgG4-related disease (IgG4-RD), but the relationship between the diseases is uncertain [62]. IgG4-RD is a non-neoplastic fibroinflammatory multisystem disease that involves lymph nodes with follicular hyperplasia and fibrosis [77]. However, lesions in IgG4-RD are distinguished by dense plasma cell infiltrates, with a significant proportion of IgG4-positive plasma cells (typically > 10 per high-power field or an IgG4/IgG ratio ≥ 40%) [78]. In contrast, KD does not show a predominance of IgG4-positive plasma cells and lacks the extensive fibrosis characteristic of IgG4-RD.

## Conclusion

Kimura disease is a unique inflammatory condition that poses diagnostic and therapeutic challenges due to its rarity and overlapping features with other disorders. A thorough understanding of its clinical, histologic, and immunologic characteristics is essential for accurate diagnosis and management. While the prognosis remains favorable, further research into its pathogenesis and novel therapies may enhance patient outcomes.

## Data Availability

No datasets were generated or analysed during the current study.

## Declarations

### Statistical Declaration

No statistical analysis was performed for this manuscript.

### Disclaimer

The views expressed herein are those of the authors and do not necessarily reflect the official policy or position of Walter Reed National Military Medical Center, Fort Belvoir Community Hospital, the U.S. Army Medical Department, the U.S. Army Office of the Surgeon General, the Department of the Air Force, the Department of the Army, Department of Defense, the Uniformed Services University of the Health Sciences, or any other agency of the U.S. Government. The identification of specific products or scientific instrumentation is considered an integral part of the scientific endeavor and does not constitute endorsement or implied endorsement on the part of the authors, DoD, or any component agency.
